# Occupation-related antecedents, job-related outcomes, and intervening factors of mental health disorders among food handlers: A scoping review protocol

**DOI:** 10.12688/f1000research.150054.1

**Published:** 2024-06-07

**Authors:** Harshit Singh, Senthilkumaran Piramanayagam, K Thirugnanasambantham

**Affiliations:** 1 Welcomgroup Graduate School of Hotel Administration, Manipal Academy of Higher Education, Manipal, Karnataka, 576104, India

**Keywords:** Food handlers, Mental health disorders, Pathogenesis, Intervening variables, Occupational outcomes

## Abstract

**Background:**

Mental health and work are closely interlinked. Evidence suggests that food handlers suffer from poor mental health across the globe. A job in the food service sector is emotionally demanding as employees directly interact with customers who insist on impeccable services and real-time responses. The causes of mental health conditions among food handlers are multifactorial. Studies on the antecedents, outcomes, and intervening factors of foodservice employees’ mental health are fragmented and sparse.

**Objective:**

We aimed to explore the existing literature describing the antecedents and outcomes of mental health disorders among food handlers. Moreover, we also aimed to explore various contextual factors that directly or indirectly influence the relationship between multiple antecedents and outcomes of food handlers’ mental health.

**Inclusion criteria:**

Studies that focus on identifying the antecedents, outcomes, and contextual factors that influence the relationship between the antecedents and consequences of food handlers’ mental health will be included in the scoping review.

**Methods:**

This scoping review will follow Arksey and O'Malley's (2005) framework: 1) identifying the research question; 2) identifying relevant studies; 3) study selection; 4) charting the data; and 5) collating, summarizing, and reporting results. Comprehensive searches will be conducted in databases such as PubMed, PsycINFO, and CINAHL using relevant keywords. Studies meeting the inclusion criteria that focused on antecedents, outcomes, and contextual factors influencing the mental health of food handlers will be selected. Data will be extracted and charted in electronic form. The findings will be narratively summarized and thematically analyzed to answer the research questions.

## Introduction

The prevalence of mental health disorders is high worldwide. The World Health Organization (WHO) report on Global Mental Health 2022 indicates that one in eight individuals in the world is living with mental disorders ( World Health Organisation, 2022). The WHO defines mental health as “ *A state of mental well-being that enables people to cope with the stresses of life, to realize their abilities, to learn well and work well, and to contribute to their communities. Mental health is an integral component of health and well-being and is more than the absence of mental disorder.*” The WHO emphasizes that mental health is a basic human right and an integral element of our general health and well-being ( World Health Organisation, 2022). The prevalence of mental health conditions and their socioeconomic consequences is enormous. A health condition is a broad term that generally covers mental and psychological disorders.

The cost of mental condition to the world economy, which stood at approximately US$ 2.5 trillion in 2010, is expected to reach US$ 6 trillion by 2030, alongside an increase in social cost. Low- and middle-income countries (LMIC) alone need to bear 35 per cent of the total cost of healthcare for mental health conditions. In addition to the direct costs involved in the treatment of mental health conditions, countries also face indirect costs, such as reduced economic productivity, unemployment, societal inequality, suicides, and substance use. In most societies, mental health is neglected and fails to provide care and support to people ( World Health Organisation, 2022). Although mental health conditions are common across the globe, millions of individuals suffer from silence, which has a significant negative impact on their daily lives. The World Mental Health Report published by the WHO in 2022 indicates that even after publishing its landmark health report in 2001, the recommendations remain valid today ( World Health Organisation, 2022).

Mental health and work are also highly interlinked. Employees’ mental health is a ubiquitous concern in the workplace. While most employees report at least one symptom of poor mental health, approximately 20 per cent of employees have mental illness ( Rosado-Solomon et al., 2023). Employees’ mental health has a huge impact on organizations and, subsequently, on global society. A collective policy brief on mental health by the WHO and the International Labour Organization (ILO) indicates that 12 billion working days are lost every year due to anxiety and depression ( World Health Organisation & International Labour Organisation, 2022). Poor mental health can influence an individual employee’s physical health, which further increases the risk of accidents, poor quality of work, and higher levels of absenteeism. Good mental health among employees enables them to be more productive, and vice versa, in unhealthy work environments. Individuals are able to function, cope, connect, and thrive better because of their good mental health ( World Health Organisation & International Labour Organisation, 2022).

Food handlers can be anyone who directly or indirectly handles unpackaged and packaged food, food equipment, and utensils used to serve or prepare food ( Food and Agriculture Organization of the United Nations, 2017). Food handling is the most common occupation and is exposed to many stress factors. The management expectation of dedication and commitment, which ultimately leads to a long working hours culture, makes food service employees have a higher average risk of exposure to depression, stress, anxiety, and chronic pain ( Cerasa et al., 2020). Moreover, studies have shown that verbal aggression, bullying, physical aggression, screams, and threats are common in the food service workplace ( Cristina et al., 2021). The existing literature on mental health-related challenges among food handlers provides a diverse set of factors that are associated with mental health. Researchers have also highlighted that events such as global health emergencies like the COVID-19 pandemic have fuelled mental health challenges among food handlers. For example, perceived stress has been found to increase owing to occupation-related stressors among food service employees during the pandemic ( Wilkesmann & Wilkesmann, 2021).

**Figure 1.**
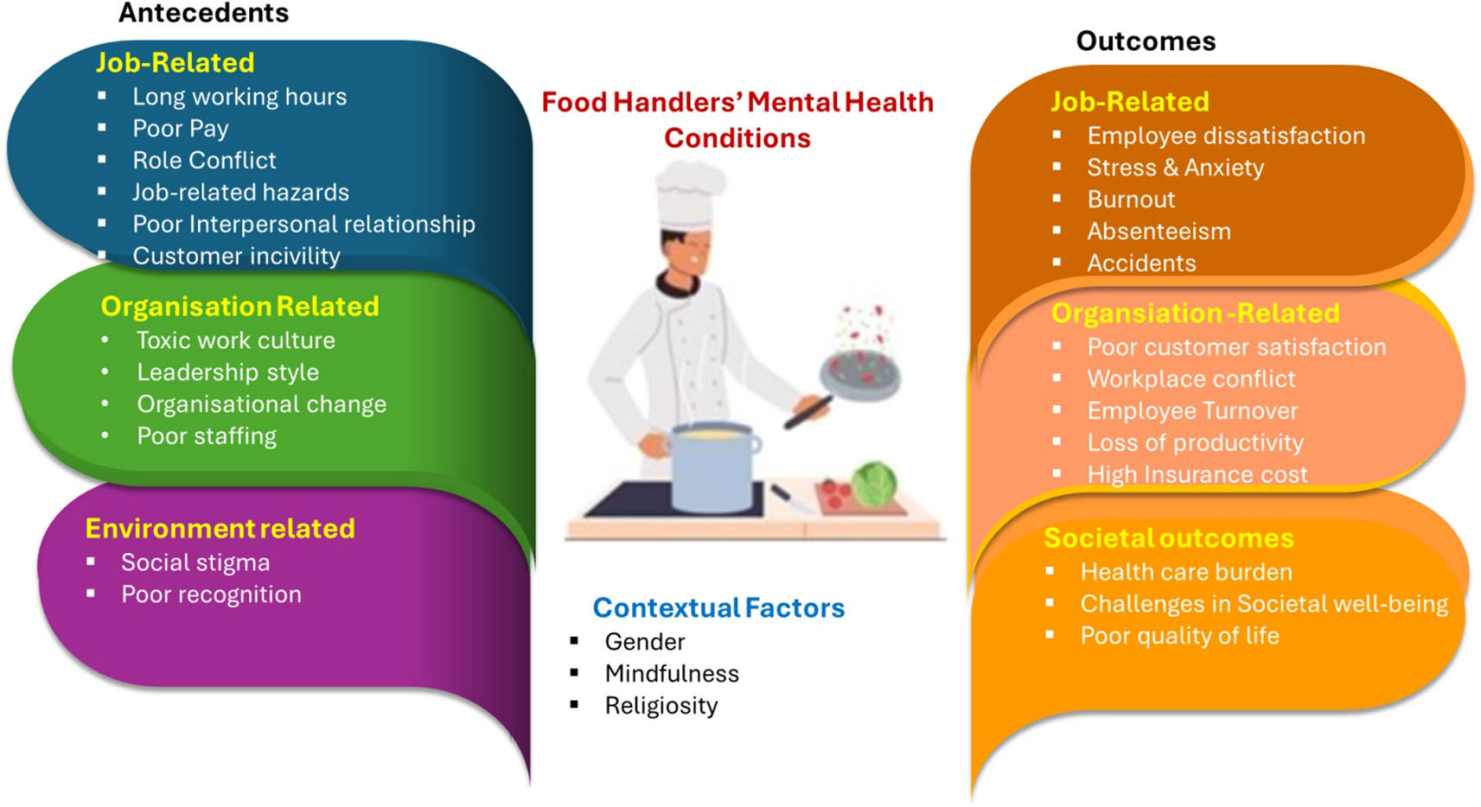
Food Handler’s mental health conditions.

Research studies that attempt to identify the factors that are critical for mental health among food handlers are multifactor: job-related factors ( Eburne & Eburne, 2010; Ishaque et al., 2021) person- or employee-related factors ( Chuang et al., 2011; Harris & Giuffre, 2010; Hinterstoisser, 2011), organization-related factors ( Chen & Wang, 2019; Chuang et al., 2011; Ishaque et al., 2021), contextual factors ( Cerasa et al., 2020; Cristina et al., 2021; Harris & Giuffre, 2010; Hinterstoisser, 2011; Kohli & Mehta, 2022) and external environment-related factors ( Chen & Wang, 2019; Wilkesmann & Wilkesmann, 2021). However, there is no synthesized evidence on the antecedents of mental health conditions among foodservice employees. In a similar manner, outcomes of mental health conditions also described in the literature. It is also multidimensional, as the mental health of food handlers has significant effects on individuals, organizations, family members, and society at a large ( Cerasa et al., 2020; Chen & Wang, 2019; Chuang et al., 2011; Cristina et al., 2021; Harris & Giuffre, 2010; Hinterstoisser, 2011; Ishaque et al., 2021; Kohli & Mehta, 2022; Pidd et al., 2015). The most common outcome frequently highlighted in literature is employee turnover. The most common antecedents, outcomes, and contextual factors are presented in Figure 1.

While employee mental health and well-being are crucial, there remains a dearth of research in this area, particularly among foodservice handlers ( Cristina et al., 2021; Ishaque et al., 2021; Kohli & Mehta, 2022). Our objective was to fill this gap by conducting a thorough scoping review that delves into the antecedents, outcomes, and intervening factors associated with mental health disorders among food handlers, as detailed in this protocol.

## 2. Methods

The researchers in the proposed scoping review will adopt the updated methodological guidelines of the JBI for the scoping review protocol ( Peters et al., 2020). A preliminary search of PROSPERO, MEDLINE, JBI Evidence Synthesis, and Cochrane Database of Systematic Reviews was conducted to confirm that no current or scoping reviews or systematic reviews were underway.

This review aims to examine the factors that contribute to mental health conditions among food handlers worldwide. We will identify the factors associated with mental health conditions, including stress, depression, anxiety, and burnout, among food handlers. These factors are classified as individual- or person-related, occupation- or work-related, organizational-, and environment-related. We will also explore the outcomes of mental health conditions among food handlers in individuals, organizations, and society. We will look at potential mediators and moderators that may influence the complex relationship between the factors that influence foodservice employees’ mental well-being. Through a comprehensive review of the literature, we hope to highlight gaps, synthesize insights, and inform future research, practices, and policies to effectively address mental health concerns among food handlers, who are an overlooked occupational group. The research questions of the proposed scoping review were developed based on Population, Concept and Context (PCC framework). The framework is presented in Table 1.

**Table 1.** PCC Framework of the proposed scoping review.

P	Population	Chefs/cook/gastronomes/Kitchen workers/waiters
C	Concept	Mental Health/Stress/Anxiety/Burnout/Occupational stress
C	Context	Restaurants/food service industries/street food vending/institutional catering

With the above PCC framework, we aimed to address the following research questions: (1) What are the occupation-related factors that cause mental health conditions in food handlers? (2) What are the outcomes of food handlers’ mental health conditions for individuals, organizations, and society? (3) Do any intervening factors moderate or mediate the relationship between antecedents and outcomes of food handlers’ mental health? (4) What strategies have been adopted to address mental health conditions among food handlers?

### 2.1. Eligibility criteria

Studies that are considered potential to be included in the scoping review, if it: (i) associated with food handlers including chef, cook, waiter, gastronomes, and workers associated with food handling experiencing the metal health conditions and its impact on individuals, organizations, and society; (ii) published in English; (3) full text; (4) adopted quantitative, qualitative, or mixed-method study designs; and (5) grey literature including conference proceedings, presentations, and posters. The authors will be contacted if only the abstract of the study is available. Research works published in other languages and the non-availability of full text that studied mental health associated factors will be excluded.

### 2.2. Search strategy

The search approach aims to locate both published and unpublished studies. An initial limited search across Web of Science, Scopus, Google Scholar, PubMed, EMBACE, and CINAHL was conducted to identify key articles, extract relevant keywords, and index terms describing the topic. With guidance from a library specialist, a comprehensive search string was developed by combining these terms using Boolean operators. I). The search terms and string used in preliminary search strategy for PubMed is presented in Table 2.

**Table 2.** Preliminary search strategy for PubMed.

Food Service establishments	((((((restaurants) OR (“food service establishment”)) OR (“food processing units”)) OR (canteens)) OR (Street food vendors)) OR (“food courts”)) OR (“food establishments”))
AND	
Mental health	((((((Psychological wellness), (Cognitive health)) OR (Mindful health)) OR (Behavioural health)) OR (Psychological well-being)) OR (Mental wellness)) OR (Mental health)) OR (Emotional stability)) OR (Psychosocial health)) OR (Inner balance)) OR (Stress)) OR (Burnout)) OR (Anxiety)) OR (Occupational stress)) OR (Cognitive functioning)) OR (Mental resilience))
Food Handlers	(((((Chefs) OR (Cooks)) or (Street Vendors)) OR (Food Hawkers)) OR (Food Helpers)) OR (Gastronomes) OR (Waiters) OR (Kitchen Workers) OR (Restaurant Operators)) OR (Servers)) OR (Stewards))
Filters	From 2000-2024

This search will be adapted for each database/information source included: PubMed, EMBASE, CINAHL, Web of Science Core Collection, PsycINFO, and ProQuest Dissertation and Theses Global for unpublished literature. The reference lists of all the included evidence sources will also be handsearched to identify additional eligible studies. The search strategy utilized a combination of controlled vocabulary (e.g., MeSH, CINAHL Headings) and natural language terms related to the population (food handlers), concept (mental health), and context (workplace). Proximity and truncation operators were applied to capture the relevant variations in terminology.

### 2.3. Selection of studies

Our search will cover all relevant databases to generate a list of citations that will be organized and uploaded onto a systematic review platform. To ensure accuracy, duplicate records will be removed during the uploading process. After training with a pilot sample, two independent reviewers screen the titles and abstracts of the remaining citations to assess their eligibility. Citations that meet these criteria will be subjected to a full-text review. Both reviewers will evaluate the full text of these citations against the inclusion criteria, and any reasons for exclusion will be recorded in detail. In cases of disagreement between the reviewers, a third reviewer will be consulted to resolve the issue.

### 2.4. Data extraction

Two independent reviewers will extract data from all the studies included in the scoping review using a standardized electronic data extraction form. The form will be developed specifically for this review to capture relevant details about the participants, concepts, contexts, study methods, and key findings related to the research questions. The data to be extracted from the existing literature is presented in Table 3.

**Table 3.** Data extraction tool.

Item	Description (including examples of categories, which will be extended based on included studies)
Name of reviewer	
Citation information	
Study title	
Study ID (Authors, Publication year)	
Journal name	
Author affiliation	
Contact information	
Funding(s)	
Unit of Analysis	
Inclusion criteria (all must be present)	
Study characteristics	
Aim/Objectives of the study	
Study period	
Study settings [Regions, Countries and Context]	
Nature of Food Service Establishment [Restaurants/Food courts /Street Food vending/Canteen/Mess/Hotel]	
Study design	
Target Population	
Years of data collection	
Sample size	
Sampling method	
Characteristics of Participants (e.g. Age, Sex and Socio-economic status)	
Measurement Tool or Instrument used [Likert Scale]	
Scale of Measurement [Categorical/Scale]	
Study Perspective	
Independent Variable(s)	
Interventions	
Mediating Variables	
Moderating Variables	
Control Variables	
Statistical Tool used	
Outcomes (Individual, interpersonal, organizational, etc)	
Key Findings	
Limitations of the study	

Prior to commencing full data extraction, the two reviewers will independently pilot test the form of the three included studies to ensure clarity, comprehensiveness, and functionality. Based on their experiences during this pilot phase, the form will be revised and finalized as needed, with any modifications documented transparently. Throughout the data extraction process, the two reviewers will work independently and meet periodically to discuss and resolve any disagreements through a consensus. If disagreements persisted after discussion, a third reviewer will be consulted to achieve resolution. For any studies published within the last 24 years where critical data are missing or unclear, attempts will be made to contact the original study authors to request additional information. Online systematic review software ( https://www.cadima.info/) will be utilized to manage and streamline the data-extraction process. This systematic approach to extracting and documenting relevant data elements aims to capture key evidence from the literature in a consistent and comprehensive manner.

### 2.5. Data analysis and presentation

The extracted data will be synthesized and presented using both narrative and visual formats. If sufficient studies are available, findings related to mental health factors among food handlers will be organized into relevant thematic categories. These may include individual-level factors (e.g., socio-demographics and coping strategies), job/task characteristics (e.g., workload and emotional demands), organizational aspects (e.g., workplace policies and support systems), and broader contextual influences (e.g., cultural norms and regulatory environment). The analysis aims to systematically map the range of determinants and consequences associated with mental health conditions, such as stress, burnout, depression, and anxiety, specifically among this workforce. Potential moderating and mediating variables that impact the complex interplay between antecedents and outcomes will also be examined where the data permits. Diagnostic approaches and assessment methods used to evaluate mental health in food handler populations across studies will be summarized, highlighting potential strengths, limitations, and research gaps. Impacts on quality of life and workplace aspects like absenteeism, productivity and turnover will be narratively synthesized. Wherever feasible, the findings will be visually depicted using tables, figures, and conceptual models to provide an integrated understanding. Differences in mental health factors across relevant subgroups (e.g., occupational roles, geographical regions, workplace settings) will also be analyzed and presented.

## 3. Discussion

We plan to conduct the first scoping review that focuses on consolidating evidence across the intersecting issues of mental health conditions among food handlers, a vulnerable occupational population. This review aimed to provide a broad map of the available research landscape related to mental health status, associated factors, and relevant interventions among food handlers across diverse contexts. We will analyze the coverage, insights, and remaining knowledge gaps to guide future studies and organizational efforts to address this topic. We will use a systematic strategy to capture published and grey literature across several databases and search for citations. However, there are some limitations regarding the language (only English) and databases screened due to logistical constraints. We should note that scoping reviews do not assess the methodological quality or risk of bias in the included studies, and we will not do so either. Despite its limitations, this scoping review represents a crucial step in consolidating the current fragmented evidence on mental health among food handlers. By synthesizing available research across diverse contexts, this review will provide a comprehensive overview of the current state of knowledge, facilitating the identification of gaps and priorities for future investigations. These findings can inform the development of targeted interventions, workplace policies, and training programs tailored to the unique needs and challenges faced by food handlers, ultimately promoting their mental well-being and overall occupational health.

### Reporting guidelines

Followed PRISMA-ScR guidelines

## Data Availability

No data are associated with this article. Mendeley: Occupation-related antecedents, job-related outcomes, and intervening factors of mental health disorders among food handlers: A scoping review protocol; https://doi.org/10.17632/9972jsjsrw.1 ( Piramanayagam et al., 2024) Data are available under the terms of the CC0 1.0 UNIVERSAL license (CC0).
