# Supplementary material for: Occupational factors and mental health outcomes among professional food handlers: A scoping review protocol
Source: F1000Res. 2025 May 6;13:600. Originally published 2024 Jun 7. [Version 2] doi: 10.12688/f1000research.150054.2 (PMC12296267; doi:10.12688/f1000research.150054.2)
Supplement: Peer review history

# Reviewer report: Reviewer response for version 2

**DOI:** 10.5256/f1000research.181370.r357201

**Published:** 2025-08-23

**Authors:** Ajit Singh

**Affiliations:** 1 Chandigarh University, Sahibzada Ajit Singh Nagar, Punjab, India

**1. Figure 1** is referenced but not fully described or critically discussed. Recommendation: Provide more explanation of the visual in the text and ensure it’s professionally designed and readable.

**2. Inclusion and Exclusion Criteria**: While types of studies are listed, the criteria are somewhat vague. There’s no explanation of how grey literature will be evaluated. **Recommendation**: Elaborate on inclusion/exclusion criteria, especially regarding grey literature (e.g., how presentations/posters will be located and assessed), clarify if only peer-reviewed studies will be included or if dissertations and government reports are also acceptable.

 3. The authors has  frequently used first-person pronouns such as *"We plan,"* *"We will analyze,"* and *"We will use."* ( Refer discussion section ). While this style is acceptable in some journal formats, it is generally advisable particularly in formal research protocols and scientific manuscripts to adopt a more objective and impersonal tone. The repeated use of first-person language may reduce the perceived formality and scientific neutrality of the work.

 For better alignment with academic writing conventions and to enhance the scholarly tone of the manuscript, it is recommended that the authors revise these statements using passive or third-person constructions. For example:

- Instead of: *"We will analyze the coverage, insights, and remaining knowledge gaps...* **"**
  Use: *"The study aims to analyze the coverage, insights, and remaining knowledge gaps..."*
- Instead of: *"We will use a systematic strategy..."*
  Use: *"A systematic strategy will be used..."*

Is the study design appropriate for the research question?

Partly

Is the rationale for, and objectives of, the study clearly described?

No

Are sufficient details of the methods provided to allow replication by others?

Yes

Are the datasets clearly presented in a useable and accessible format?

Yes

Reviewer Expertise:

Area of Research : Hospitality , Tourism , and Management.

I confirm that I have read this submission and believe that I have an appropriate level of expertise to confirm that it is of an acceptable scientific standard, however I have significant reservations, as outlined above.

# Reviewer report: Reviewer response for version 2

**DOI:** 10.5256/f1000research.181370.r392265

**Published:** 2025-07-26

**Authors:** G.K. Sreenivasan

**Affiliations:** 1 Bundelkhand University, Jhansi, Uttar Pradesh, India

No doubt, the food handlers actually go through severe work stress due to increasing number of competitors, expectations of the guests and work performance upto the satisfaction of the co-employees and employers.  Therefore, the researchers deserve a mark of appreciation.  The introduction cum literature review provides a proper base to the study.  Further, consequently, appropriate objectives are identified.  

 The objectives seem to be achievable easily with the research design provided.  

 From out of the summary of the major revisions given, it is apparent that the authors have seriously followed the review undertaken by the first reviewer.  

 No issues of competent interest are found.  

 The reviewer expects the full-length paper eagerly.

Is the study design appropriate for the research question?

Yes

Is the rationale for, and objectives of, the study clearly described?

Yes

Are sufficient details of the methods provided to allow replication by others?

Yes

Are the datasets clearly presented in a useable and accessible format?

Yes

Reviewer Expertise:

work-life balance, tourism marketing, e-marketing service marketing, customer relationship marketing

I confirm that I have read this submission and believe that I have an appropriate level of expertise to confirm that it is of an acceptable scientific standard.

# Reviewer report: Reviewer response for version 2

**DOI:** 10.5256/f1000research.181370.r383211

**Published:** 2025-06-03

**Authors:** Benta Mbare

**Affiliations:** 1 Tampere University, Tampere, Finland

The revised version of the manuscript is clearly written and generally easy to follow. However, I have a few concerns that could be addressed to strengthen its clarity and rigor.

***Introduction and focus***

 The first and second paragraphs of the introduction appear somewhat scattered in their review of mental health, particularly the second paragraph. While mental health is indeed an important issue, it is a broad domain, and not all mental health challenges originate from or are influenced by the work environment. The manuscript currently lacks a clear explanation of how the general discussion on mental health, along with the cited costs, directly connects to the study’s specific focus. I think the paper would benefit from narrowing the scope to occupational mental health, with an explanation to how it relates to the population under study. Alternatively, if the intention was to consider broader mental health issues (including non-work-related factors), then I think the authors should more explicitly justify how these are relevant to the study objectives.

***Use of references***

 The introduction appears to rely heavily on a single source, WHO (2022), with limited engagement with other empirical literature. While the WHO reference is important, I think the manuscript would benefit from the inclusion of additional, more recent, and diverse scholarly sources to better situate the study within the current academic discourse. Additionally, some of the cited references seem somewhat outdated and may not reflect the latest developments or findings in this area. I suggest for the authors to incorporate more recent empirical studies that align with the study’s objectives.

***Feasibility and scope of the study***

 In my opinion, the study’s aim to examine mental health among food handlers globally raises some feasibility concerns. Labour market characteristics, occupational conditions, and cultural perceptions of mental health vary significantly across countries, and these differences are likely to influence the outcomes. It is unclear whether the study intends to conduct a comparative analysis across different contexts, and if so, which elements will be examined to account for these variations. Clarification on the intended scope and methodological approach to address such disparities would be helpful in evaluating the feasibility and relevance of the intended study.

Is the study design appropriate for the research question?

Yes

Is the rationale for, and objectives of, the study clearly described?

Yes

Are sufficient details of the methods provided to allow replication by others?

Partly

Are the datasets clearly presented in a useable and accessible format?

Not applicable

Reviewer Expertise:

Health and development; occupational health; organisational psychology, inequalities and inclusions, minorities, health science; qualitative research

I confirm that I have read this submission and believe that I have an appropriate level of expertise to confirm that it is of an acceptable scientific standard, however I have significant reservations, as outlined above.

# Reviewer report: Reviewer response for version 1

**DOI:** 10.5256/f1000research.164588.r299033

**Published:** 2025-04-28

**Authors:** Hala Samir Abou-ElWafa

**Affiliations:** 1 Industrial Medicine and Occupational Health, Public Health & Community Medicine Department, Mansoura University, Mansoura, Dakahlia Governorate, Egypt

- The magnitude of the problem of mental health disorders among food handlers was not clearly stated in order to justify carrying out this review. So, the burden of the problem should be more elucidated otherwise it will not be of public health importance and not deserving further reviewing.
- Searching for literature from year 2000-2024 is a very long duration. I suggest being more concise for example in the last 10 years.
- Authors mentioned that grey literature will be searched too including presentations and posters also but did not clarify how you will find them and their weight of evidence.

Is the study design appropriate for the research question?

Yes

Is the rationale for, and objectives of, the study clearly described?

Partly

Are sufficient details of the methods provided to allow replication by others?

Partly

Are the datasets clearly presented in a useable and accessible format?

Partly

Reviewer Expertise:

Occupational health and industrial medicine

I confirm that I have read this submission and believe that I have an appropriate level of expertise to confirm that it is of an acceptable scientific standard, however I have significant reservations, as outlined above.

# Reviewer report: Reviewer response for version 1

**DOI:** 10.5256/f1000research.164588.r348983

**Published:** 2025-01-02

**Authors:** Benta Mbare

**Affiliations:** 1 Tampere University, Tampere, Finland

This protocol is written concisely and is easy to follow and understand. However, the intended study appears to be very broad in scope which is too ambitious and may challenge the analysis process. Below are my reflections

**Title: **The current title of the study does not effectively convey the purpose or focus of the research. At present, it appears to be a collection of terminologies, some of which are repetitive and lack coherence. To improve clarity and impact, the title should be restructured to succinctly reflect the study's objectives and scope.

**Broader context:** Mental health is a broad and multifaceted subject. Thus, the background part should emphasise on occupational mental health and also mention the global prevalence and burden of occupational mental health conditions.

**Focus of study:** Food is handled by many professionals and consumers. It is my understanding that the authors aim to investigate food handlers specifically within professional settings. I think the title should explicitly reflect this distinction to avoid confusion as the term “food handlers” sound quite broad.

**Feasibility and scope:** The aim to study mental health among food handlers worldwide may not be entirely feasible, especially given the significant differences in labour market profiles and occupational conditions across countries. These disparities are likely to influence the findings on "mental health." Is there an intended comparative analysis regarding these distinctions, and what are the specific elements to be examined?

**Study selection criteria:** The scope of study selection is unclear. What types of studies are the authors intending to include? Will the review encompass qualitative, quantitative, or mixed-methods research? Are non-academic reports or grey literature (e.g., government publications, industry data) also considered, and if so, what criteria will be used to evaluate their inclusion? Providing more detail on the methodology and selection criteria would enhance the protocol’s transparency.

Is the study design appropriate for the research question?

Yes

Is the rationale for, and objectives of, the study clearly described?

Yes

Are sufficient details of the methods provided to allow replication by others?

Partly

Are the datasets clearly presented in a useable and accessible format?

Not applicable

Reviewer Expertise:

qualitative research, occupational health; organizational psychology, inequalities and inclusions, minorities, health sciences

I confirm that I have read this submission and believe that I have an appropriate level of expertise to confirm that it is of an acceptable scientific standard, however I have significant reservations, as outlined above.
